# The Construction of Risk Prediction Models Using GWAS Data and Its Application to a Type 2 Diabetes Prospective Cohort

**DOI:** 10.1371/journal.pone.0092549

**Published:** 2014-03-20

**Authors:** Daichi Shigemizu, Testuo Abe, Takashi Morizono, Todd A. Johnson, Keith A. Boroevich, Yoichiro Hirakawa, Toshiharu Ninomiya, Yutaka Kiyohara, Michiaki Kubo, Yusuke Nakamura, Shiro Maeda, Tatsuhiko Tsunoda

**Affiliations:** 1 Laboratory for Medical Science Mathematics, RIKEN Center for Integrative Medical Sciences, Yokohama, Japan; 2 Department of Environmental Medicine, Graduate School of Medical Sciences, Kyushu University, Fukuoka, Japan; 3 Department of Medicine and Clinical Science, Graduate School of Medical Sciences, Kyushu University, Fukuoka, Japan; 4 Laboratory for Genotyping Development, RIKEN Center for Integrative Medical Sciences, Yokohama, Japan; 5 Human Genome Center, Institute of Medical Science, The University of Tokyo, Tokyo, Japan; 6 Laboratory for Endocrinology, Metabolism and Kidney Diseases, RIKEN Center for Integrative Medical Sciences, Yokohama, Japan; New Jersey Institute of Technology, United States of America

## Abstract

Recent genome-wide association studies (GWAS) have identified several novel single nucleotide polymorphisms (SNPs) associated with type 2 diabetes (T2D). Various models using clinical and/or genetic risk factors have been developed for T2D risk prediction. However, analysis considering algorithms for genetic risk factor detection and regression methods for model construction in combination with interactions of risk factors has not been investigated. Here, using genotype data of 7,360 Japanese individuals, we investigated risk prediction models, considering the algorithms, regression methods and interactions. The best model identified was based on a Bayes factor approach and the lasso method. Using nine SNPs and clinical factors, this method achieved an area under a receiver operating characteristic curve (AUC) of 0.8057 on an independent test set. With the addition of a pair of interaction factors, the model was further improved (p-value 0.0011, AUC 0.8085). Application of our model to prospective cohort data showed significantly better outcome in disease-free survival, according to the log-rank trend test comparing Kaplan-Meier survival curves (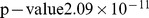). While the major contribution was from clinical factors rather than the genetic factors, consideration of genetic risk factors contributed to an observable, though small, increase in predictive ability. This is the first report to apply risk prediction models constructed from GWAS data to a T2D prospective cohort. Our study shows our model to be effective in prospective prediction and has the potential to contribute to practical clinical use in T2D.

## Introduction

The prevalence of diabetes mellitus is increasing and it has become one of the major global diseases, the most common form worldwide being type 2 diabetes (T2D) [1]. The incidence of T2D has been increasing rapidly in many countries, including Japan, over the past few decades [2]–[4] and it is estimated that 500 million individuals will be affected by some form of diabetes by 2030 if no preventive strategies are implemented [5]. The prompt establishment of a reliable risk prediction model is required as there is evidence the progression of T2D can be largely prevented by diet control and exercise if the risk (the probability of T2D) can be estimated beforehand [6].

Multiple genetic and clinical risk factors are expected to contribute to the pathogenesis of T2D. Previous studies have shown that clinical risk factors such as age, gender, body mass index (BMI), family history of T2D, systolic blood pressure, high-density lipoprotein cholesterol level, triglycerides level, insulin secretion and fasting plasma glucose are risk predictors for T2D [7], although their predictive ability may have been influenced by study design and population [8]. More recently, genome-wide association studies (GWAS) have identified and validated single nucleotide polymorphisms (SNPs) associated with T2D. *TCF7L2*, *KCNJ11*, *PPARG*, *CDKAL1*, *IGF2BP2*, *CDKN2A/2B*, *FTO* and *HHEX* are well known genetic risk factors for T2D [9]–[12]. *KCNQ1, C2CD4A/4B, UBE2E2* and *ANK1* have recently been reported as common susceptibility loci for T2D in the Japanese population [13]–[15].

There have been numerous studies developing risk prediction models using genetic risk factors [16]–[19]. However, risk prediction models using only genetic risk factors have shown a relatively low ability to predict the development of T2D compared to models composed of clinical risk factors. This is due to the low effect size of each genetic risk factor for common diseases, with per allele odds ratios ranging from 1.05 to 1.35 [20]. Even when risk prediction models were constructed using a combination of both genetic and clinical risk factors, marginal or no increase in the predictive ability was observed [8]. However, most studies have only focused on the genetic factors associated with T2D that reached GWAS significance and have not investigated if additional genetic risk factors and regression methods are useful for model construction. In this study, we examine the best risk prediction approach by applying not only algorithms for the genetic risk factor detection but also multiple regression methods for model construction.

One way to improve predictive power is to construct a risk model using more SNPs. Penalized regression methods such as ridge regression [21], elastic net [22] and lasso [23] are increasingly used in high-dimensional settings. The advantage of these approaches is they simultaneously carry out variable selection in regression models, and provide estimates of the coefficients of the selected variables. Kooperburg *et al.* and Wei *et al.* reported that using a larger number of SNPs than those which reached GWAS significance with penalized regression methods contributed to an improvement in risk model construction for Crohn's disease [24] and inflammatory bowel disease [25].

In addition to regression methods for the model construction, several algorithms for risk factor detection have been recently proposed, such as asymptotic Bayes factors (ABF) [26] and sure independence screening (SIS) [27]. ABF can consider various choices of the prior on the effect size, including those that allow effect size to vary with the MAF of the marker. SIS can select more informative SNPs by maximization of marginal likelihood estimates using regression model. However, to our knowledge, analysis considering either these algorithms or interactions between risk factors has yet to be reported.

In this study, we applied 10-fold cross-validation to a training set of 6,624 Japanese individuals, separated from a test set of 736 Japanese individuals. We first ranked successfully genotyped SNPs using three algorithms for risk factor detection: the Cochran-Armitage for trend, ABF and SIS with an additive association model. Using risk factors composed of a combination of the top-ranked SNPs (genetic risk factors) with each set of the cross-validation and clinical risk factors (age, gender and BMI), we then constructed risk prediction models based on three penalized regression methods; the ridge regression [21], the elastic net [22] and the lasso [23]. After we constructed the model based on the entire training set, we evaluated our risk prediction model with an independent test set by the area under the receiver operating characteristic curve (AUC). Furthermore, we considered interactions between the risk factors. Finally, we evaluated the predictive ability of our model using a prospective cohort.

## Results

### Association studies in Japanese individuals

**Figure 1:**
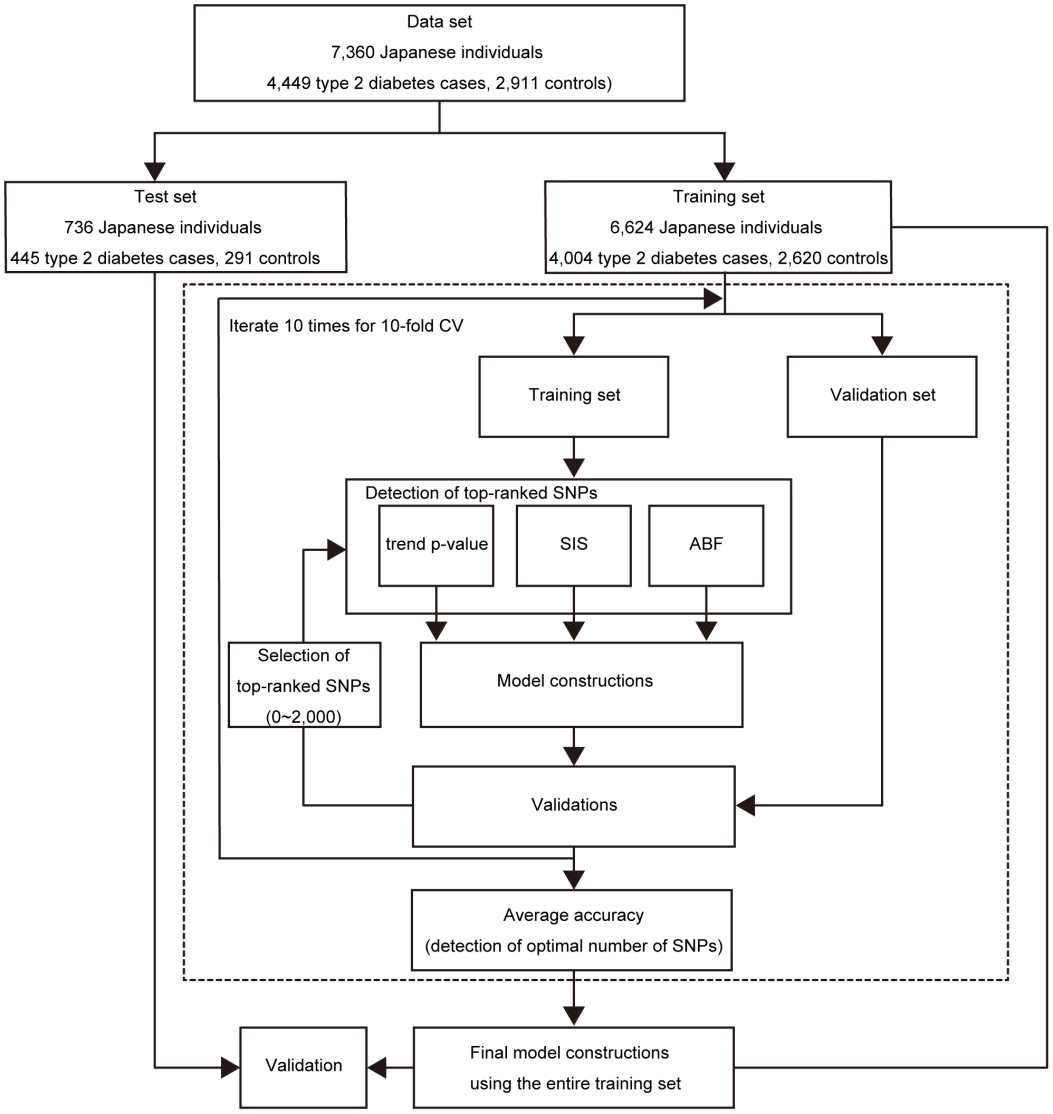
Outline of the risk prediction model construction and validation.

**Figure 2:**
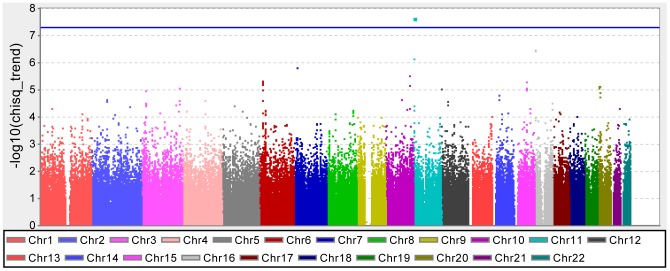
Results of whole genome association scan for a training set.

We separated 7,360 Japanese individuals (4,449 type 2 diabetes cases, 2,911 controls) into a training set of 6,624 individuals (4,004 T2D cases, 2,620 controls) and a test set of 736 individuals (445 T2D cases, 291 controls) (see Materials and Methods, Figure 1). The quantile-quantile (QQ) plots of the p-values from the Cochran-Armitage test for trend showed the genomic inflation factor 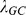 to be 1.06 (Figure S1) [28]. Only one locus, *KCNQ1*, which has been previously reported to be associated with T2D in Japanese and European populations [13], reached a genome-wide significance level (rs2237892; 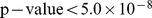, Figure 2).

### Construction of risk prediction models

Selection of risk prediction models was performed using 10-fold cross-validation on the training set (Figure 1). In addition to the detection of the most significant SNPs using the three algorithms described above, we considered cases with and without taking into consideration the linkage disequilibrium (LD) between two SNPs (see Materials and Methods).

**Figure 3:**
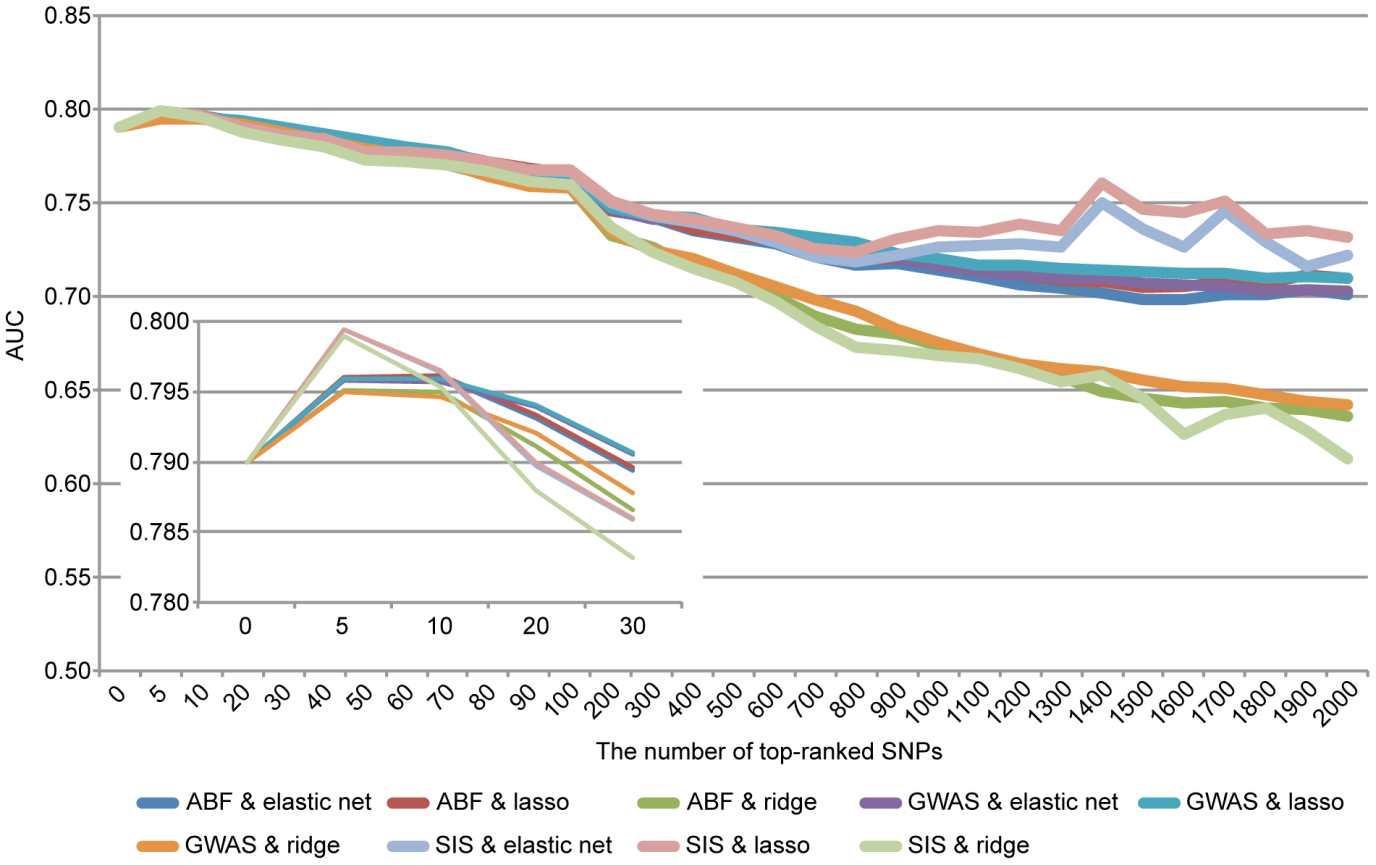
Risk prediction models using 10-fold cross-validation on the training set.

**Figure 4:**
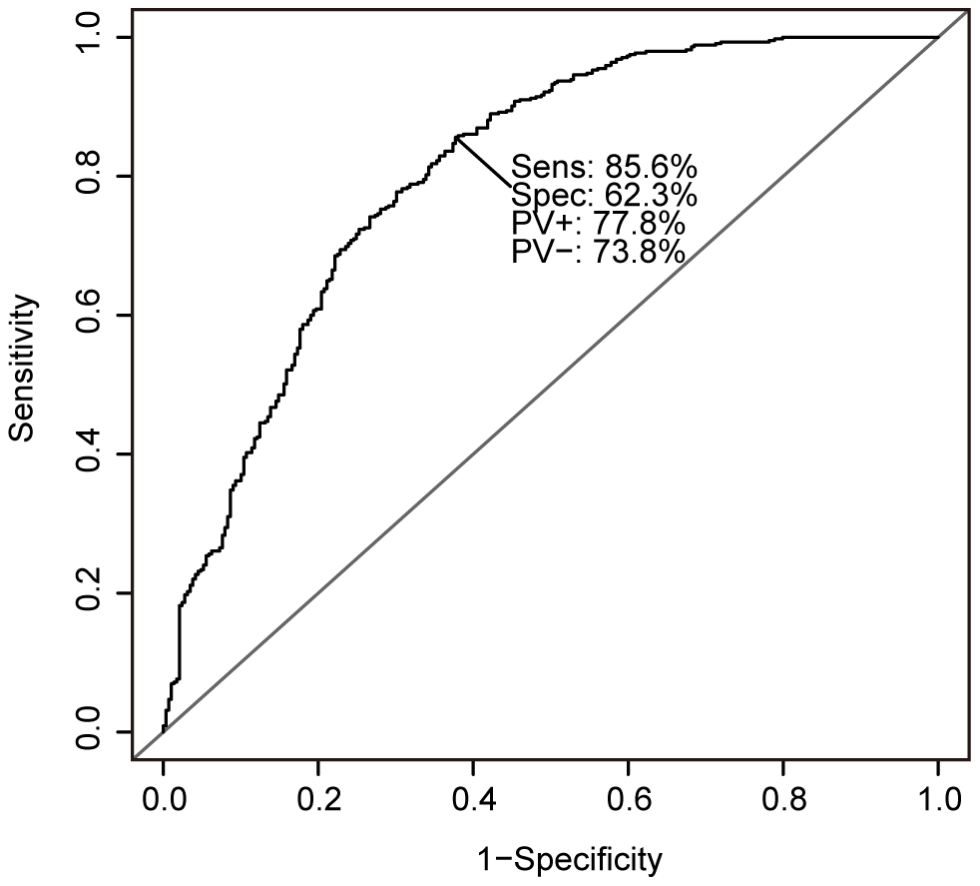
The ROC curve for our risk prediction model. Sensitivity and specificity was maximized at a sensitivity of 0.858 and specificity of 0.623.

All approaches that we considered were carried out on data sets of the *p* most significant SNPs in a stepwise manner (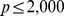). Nine-tenths of entire training set of 6,624 individuals was used to determine the most significant SNPs (top-ranked SNPs) using the Cochran-Armitage test for trend, ABF and SIS with an additive association model and to fit the model for each cross-validation step. The adjusted model was evaluated using the remaining one-tenth of the training set. For model construction, we applied penalized regression methods: the ridge regression, elastic net and lasso methods. This process was repeated 10 times (10-fold cross validation). On the basis of the average AUC, we determined the optimal number of SNPs for model construction for each combination of algorithms and methods. For both the Cochran-Armitage test for trend and SIS, the highest AUC was observed when the top 5 SNPs were selected for model construction (Figure 3). In the case of ABF, the highest AUC was observed using the top 10 SNPs (Figure 3). Final models were constructed using the complete training set and the determined top-ranked SNP count (Figure 1). The adjusted models were then evaluated on the test set, which was completely independent from the training set. When top-ranked SNPs were detected with ABF considering the information of linkage disequilibrium between SNPs, and the risk models were constructed based on the lasso method, the highest AUC of 0.8057 was achieved, which was greater than that of the model constructed using only clinical risk factors (Table 1). A maximum average sensitivity and specificity of the ROC curve was achieved at a sensitivity of 0.858 and specificity of 0.623 (Figure 4). In order to show the range of effect sizes for this model, we further randomly separated the entire data into a training set and a test set, in a nine to one ratio, 200 times. We constructed the model based on the top 10 SNPs determined above and the lasso method using the training set. We evaluated the model on the independent test set with respect to each split. The range of effect sizes was obtained from the minimum and maximum of the 200 AUC differences between the model with and without the genetic factors in the test set. The observed effect sizes were between 0.00129 and 0.0265 in AUC (AUC-clinical-only: 0.7562 to 0.8289, AUC-combined: 0.7601–0.8372). This result was also reflected in the analysis of a prospective cohort study (see Materials and Methods). Our model was superior to that constructed using only clinical risk factors (1.5% increase in AUC). Furthermore, we also applied support vector machines with RBF kernels to our data, which were performed using the package *kernlab* in R. The parameter sigma in SVM with RBF kernel was then optimized by 10-fold cross-validation. However, the model based on RBF kernels did not contribute to an increase in the predictive ability even when more genetic factors were used (AUC-clinical-only 0.8535, AUC-combined 0.8507) although the AUC scores were high. However, when this result was reflected in the analysis of a prospective cohort study (larger sample size collected from entirely different cohort), the AUC score was much lower than that of our regression model (AUCs 0.6072 in SVM and 0.6545 in L1-regression). The models based on RBF kernels may be overfit to the training data compared to the regression models. This result suggests that validation of risk models should be applied to at least two independent data sets, including that collected from entirely different cohort. Ideally, at least one prospective cohort study should be used to check any potential selection bias in GWAS. Similar results were observed when the dataset was randomly split into a different training and test set (data not shown).

**Table 1:** The top AUCs observed in regression methods and the number of SNPs used in risk prediction model construction.

algorithm	method	#SNPs used	AUC:clinical (95%CIs)	AUC:combined (95%CIs)
GWAS	ridge regression	5	0.7986 (0.7646–0.8326)	0.8019 (0.7682–0.8356)
	elastic net	5	0.7984 (0.7644–0.8323)	0.8025 (0.7689–0.8361)
	lasso	5	0.7984 (0.7645–0.8324)	0.8027 (0.7691–0.8363)
with r-square	ridge regression	5	0.7986 (0.7646–0.8326)	0.8019 (0.7682–0.8356)
	elastic net	5	0.7984 (0.7644–0.8323)	0.8025 (0.7689–0.8361)
	lasso	5	0.7984 (0.7645–0.8324)	0.8027 (0.7691–0.8363)
SIS	ridge regression	5	0.7986 (0.7646–0.8326)	0.7989 (0.7651–0.8328)
	elastic net	5	0.7984 (0.7644–0.8323)	0.7994 (0.7656–0.8332)
	lasso	5	0.7984 (0.7645–0.8324)	0.7995 (0.7657–0.8333)
with r-square	ridge regression	5	0.7986 (0.7646–0.8326)	0.7989 (0.7651–0.8328)
	elastic net	5	0.7984 (0.7644–0.8323)	0.7994 (0.7656–0.8332)
	lasso	5	0.7984 (0.7645–0.8324)	0.7995 (0.7657–0.8333)
ABF	ridge regression	10	0.7986 (0.7646–0.8326)	0.8050 (0.7715–0.8386)
	elastic net	10	0.7986 (0.7646–0.8326)	0.8054 (0.7719–0.8388)
	lasso	10	0.7984 (0.7645–0.8324)	0.8054 (0.772–0.8389)
with r-square	ridge regression	10	0.7986 (0.7646–0.8326)	0.8051 (0.7717–0.8385)
	elastic net	10	0.7984 (0.7644–0.8323)	0.8056 (0.7723–0.8389)
	**lasso**	**9**	**0.7984 (0.7645–0.8324)**	**0.8057 (0.7724–0.839)**

For the elastic net alpha is set to 0.5. Alphas of 0.1 to 0.9 at 0.1 intervals were tested and the complete results are in Table S2. Many coefficients of the lasso and the elastic net methods are set to 0 due to variable selection in regression models.

The lasso method carried out effective selection of SNPs (Table 1). Of the 10 top-ranked SNPs, the lasso method used 9 for our risk prediction model (Table S1). The one excluded SNP, rs12922855, located on intron region of a gene *A2BP1*, was not included in the HapMap linkage disequilibrium data for the Japanese population. However, we found this SNP to be in high LD with another top-ranked SNP rs11865230 (r-squared 0.99) and thus should have been previously excluded.

### Effective SNPs used in risk prediction model construction

The nine SNPs used in our risk prediction model were located near or within 7 genes: *KCNQ1* (rs163171), *DGKB* (rs11514706), *TCF7L2* (rs7901695), *CDKAL1* (rs2328531, rs2206734), *C2CD4A/B* (rs1436953), *A2BP1* (rs11865230), and *PAK7* (rs4813894). *KCNQ1*, *DGKB*, *TCF7L2*, *CDKAL1* and *C2CD4A/B* have been previously reported in association with T2D [9] [11] [13] [14] [29] [30]. *A2BP1*, also known as *FOX-1*, was reported as a susceptibility locus for obesity [31] and *PAK7* was reported to play important roles in the pleiotropic effects on lipid metabolism and metabolic syndrome [32]. All SNPs used for our risk model construction were located in close proximity of genes with possible roles in the development of T2D.

### Improvement of risk prediction model by interaction factors

To further improve our risk prediction model, we considered interactions of not only genetic-genetic risk factors (GF-GF) but also genetic-clinical risk factors (GF-CF) and clinical-clinical risk factors (CF-CF). We investigated all 66 pairwise combinations (interactions) composed of 9 GFs (SNPs) and 3 CFs (age, gender and BMI) in our risk prediction model by comparing our risk prediction model with and without including each interaction (i.e., deviation from a multiplicative model). As a result, marginally significant p-values were observed for two interactions, age with gender (0.0020) and rs1436953 *C2CD4A/B* with rs11865230 *A2BP1* (0.0425) (Table 2). To show whether the combination of these two interactions is significant, we compared the risk prediction model with and without including these two interactions (likelihood ratio test, p-value 0.0011), and inclusion of these two interactions further contributed to an increase of AUC (0.8084).

**Table 2:** A list of significant interaction factors.

interaction (chromosome #, gene)		factor	p-value (ANOVA)
age	gender	CF-CF	0.0020
rs1436953 (15, C2CD4A/B*)	rs11865230 (16, *A2BP1*)	GF-GF	0.0425

*gene associated with T2D.

### Validation in a prospective cohort

**Figure 5:**
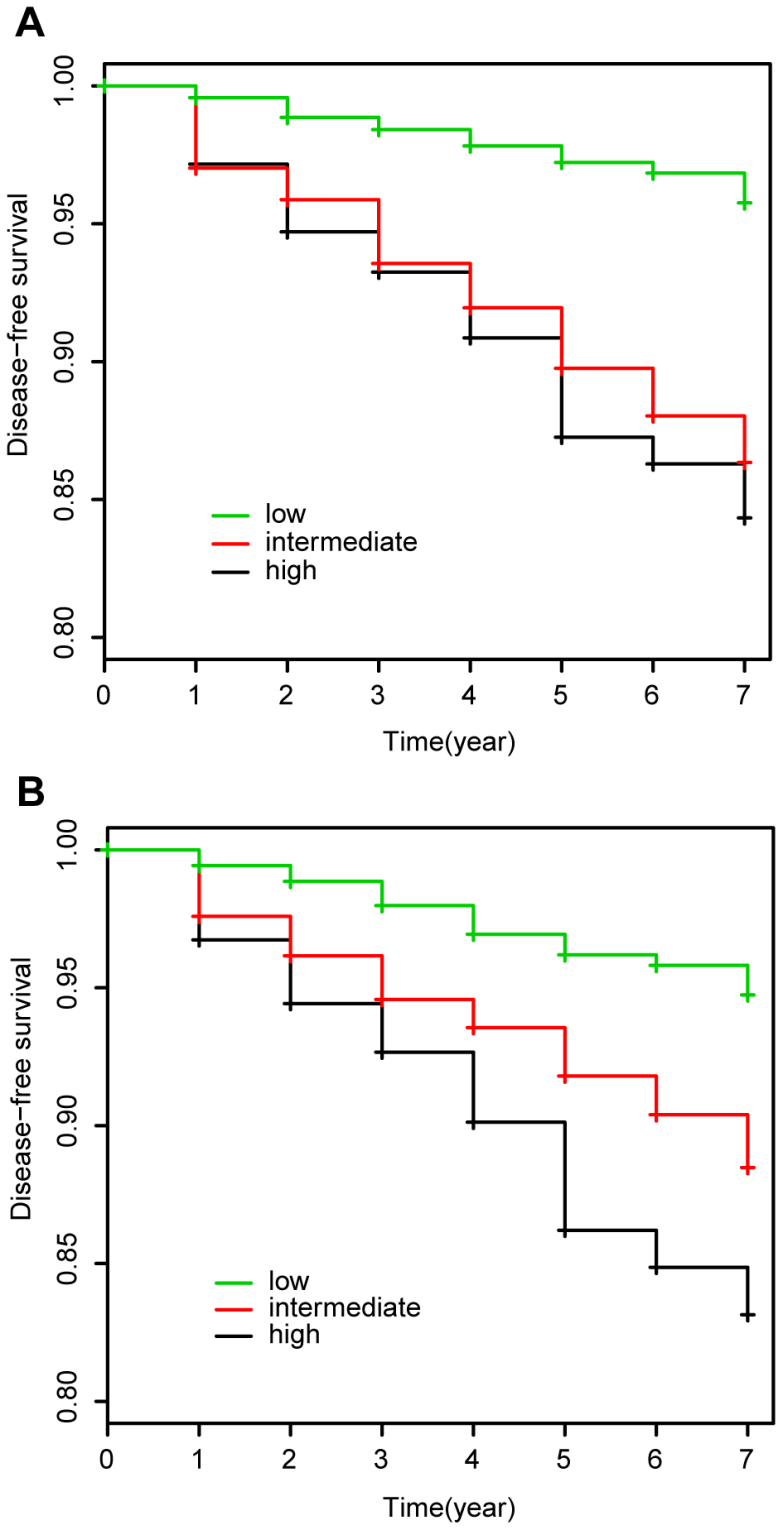
Cumulative disease-free survival in a prospective cohort. Models using (A) only clinical risk factors and (B) both of clinical and genetic risk factors.

We applied our risk prediction model to prospective cohort study data (see Materials and Methods). Risk scores assigned to each subject were calculated by applying 9 SNP genotypes and 3 clinical risk factors to our risk prediction model 
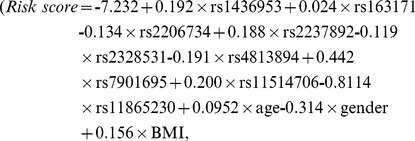
 where the regression coefficients were determined by GWAS). According to the scores, we divided the set into three equally sized risk assessment categories: high, intermediate and low. Survival probabilities were calculated using the Kaplan-Meier method. We used the KM curve because we divided prospective cohort into three equally sized risk assessment categories based on risk scores from GWAS data and the effects of the predictor variables upon survival are not constant over time (crossing hazards). We analyzed the data both considering only clinical risk factors (Figure 5A) and genetic risk factors in combination with clinical risk factors (Figure 5B). The risk prediction model that used both clinical and genetic risk factors significantly classified the three categories. The Kaplan-Meier curves showed better outcome in disease-free survival (Figure 5B, log rank trend test, 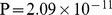) than those from only clinical risk factors (Figure 5A, log rank trend test, 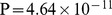). The probability of a Type II error is generally increased for log-rank and weighted log-rank tests, but we improved the performance by using *survfit* (R). While p-values for both models were extremely small, the model including genetic risk factors categorized the prospective data better than that not including genetic risk factors.

## Discussion

Owing to the success of GWAS, numerous studies have investigated the predictive ability of risk prediction models using genetic risk factors at a genome-wide significance level for T2D [16]–[19]. However, genetic risk factors have been shown to have only a small effect on a risk prediction model's ability to predict the future development of T2D as compared to those composed of only clinical risk factors [20]. One way recently proposed to improve the predictive power is to construct a risk model that uses more SNPs with smaller effect sizes associated with a phenotype. Kooperberg *et al.* and Wei *et al.* have demonstrated the usefulness of this method for Crohn's disease [24] and inflammatory bowel disease [25]. In Wei *et al.*, the final predictive models achieved AUCs of 0.86 and 0.83 for Crohn's disease (CD) and ulcerative colitis (UC), respectively. Likewise, in this study, we also suggest the usefulness of including additional SNPs for risk prediction model construction.

We constructed our risk prediction model by treating different SNPs located within the same gene as separate genetic risk factors (i.e. *KCNQ1* and *CDKAL1*), as opposed to treating such SNPs as a vector for the count of haplotypes [33]. We were interested in how this decision affected the predictive ability of our model. We estimated haplotype frequencies with the program SNPHAP [34] and constructed haplotypes for frequencies >1%. Although the predictive ability was slightly less than that of our risk prediction model (AUC 0.8054), we expect that this approach would be more effective in studies where many genetic risk factors located within or near the same genes are used for the construction of the risk model. This is because haplotypes, which are specific combinations of nucleotides on the same chromosome, can provide more information on the complex relationship between DNA variation and phenotypes than any single SNP.

We added known, previously reported SNPs associated with T2D along with our 9 identified SNPs for model construction, and conducted a risk model reconstruction using lasso method. Since we observed that the lasso method carried out effective selection of SNPs, if our 9 SNPs are essential factors for risk model construction, they should also be reselected during this model reconstruction. We used 10 previously known SNPs, that were included in our genotype data (rs1470579, rs2383208, rs1111875, rs7923837, rs5015480, rs13266634, rs8050136, rs10946403, rs6780569, rs7172432) [9] [10] [12] [14] and passed linkage disequilibrium consideration between two SNPs. In total, 19 SNPs were used for model reconstruction. The model with the ridge regression method achieved an AUC score of 0.8090. Of them, 17 were chosen by the lasso method for use in the risk model reconstruction, achieving an AUC score of 0.8095. As expected, all 9 of our SNPs were selected as essential factors for risk model construction. Although only one SNP reached a genome-wide significance level in this study, all 9 SNPs used for our risk model construction were located in close proximity of genes with possible roles in the development of T2D. This result implies our approach has the potential to identify genetic risk factors associated with disease even when the sample size of the case-control study design is relatively small. Furthermore, as a relatively small number of SNPs was sufficient for T2D risk prediction model construction, our model could contribute to an increase in cost-effectiveness of clinical genetic testing.

Interactions among genetic and/or clinical factors, which may have stronger effects in combination, are expected to increase the power of risk prediction models [35] and advance our understanding of the underlying biology [36]. We experimented with the inclusion of these interaction factors in our risk prediction models and, as a result, found two interactions that contributed to an increased AUC. In particular, the GF-GF interaction between A2BP1 and C2CD4A/B is interesting as A2BP1 has not been previously reported to be associated with T2D. In this study, we found interaction factors contributed to an increased AUC from genetic and clinical factors used for our model construction. However, we could not consider all interactions between genetic and clinical risk factors as that number would be 2,005,003 (
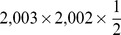
) interactions. For each additional interaction considered, the number of the combinations will increase exponentially. After correction for multiple testing, the cut-off p-value for significance becomes even so small that we would likely not find any significant interactions. Therefore, we attempted to look for significant interactions after feature selection. By comparing our risk prediction model with and without the inclusion of each interaction, the contribution to an increase in predictive ability for both interactions was found to be statistically significant. Furthermore, by comparing the risk prediction model including the two AUC increasing interactions with those including two interactions randomly selected from the genetic and clinical risk factors, we verified the combination of these two interactions is statistically significant. These results suggest that interactions of genetic/clinical risk factors can contribute to an improvement of risk prediction model and are worth considering when constructing a risk prediction model.

It has been reported that case-control study designs and population characteristics may affect the observed predictive ability of risk models. In particular, AUC values of risk models using only clinical risk factors are different even though the same clinical risk factors are used [8]. This difference was observed in both Van Hoek *et al.* [18] and Sparso *el al.* [19], as well as our study (AUCs; 0.68, 0.93 and 0.80) and is suspected to be caused by mismatches in age and BMI between the case and control sets (mean age, case/control: 68.2/69.0 (Van Hoek *et al.*), 60.0/47.0 (Sparso *el al.*), and 65.5/51.6 (our study); mean BMI, case/control: 28.0/26.0, 30.6/25.6 and 23.8/23.2, respectively). Therefore, we further applied our risk prediction model to a prospective cohort in order to verify its predictive ability. The Kaplan-Meier curves showed a much better outcome in disease-free survival than those from only clinical risk factors. Our model considering genetic risk factors contributed to a small increase in predictive ability compared with that from only clinical risk factors. This result suggests that the genetic risk factors also play a key role in the clarification of risk group category, in particular, the classification between intermediate risk and high risk (Figure 5). This clear classification of individuals will be helpful for future practical use in healthcare.

We constructed our risk prediction model by considering algorithms for genetic risk factor detection and regression methods for model construction. However, the major contribution was from clinical factors rather than the genetic factors. Given the current sample size, the contribution from genetic risk factors to the risk prediction of T2D may be limited even though we considered several algorithms and regressions. However, although small, considerations of genetic risk factors contributed to a measurable increase in predictive ability. In future work, we will perform further replication of this analysis, and investigations with larger sample sizes may lead to a greater improvement in the performance of risk prediction models.

While we propose a methodology that finds the best model for this study rather than a general model that could be applied to any data set, further refinement of this methodology will be required before its practical use in healthcare. One way may be to consider rare variants for T2D. The recent development of next generation sequencing technology has facilitated comprehensive analysis of low frequency variants. There is no doubt that additional loci would contribute to further improvement of risk prediction models.

## Materials and Methods

### Ethics Statement

This study was approved by the ethics committee of the Institute of Physical and Chemical Research (RIKEN). The design and performance of current study involving human subjects were clearly described in a research protocol. All participants were voluntary and would complete the informed consent in written before taking part in this research.

### Genotype Samples

We selected case-control samples from the subjects enrolled in the BioBank Japan. The subjects were recruited from several medical institutes in Japan, including Fukujuji Hospital, Iizuka Hospital, Iwate Medical University School of Medicine, National Hospital Organization Osaka National Hospital, Nihon University, Nippon Medical School, Osaka Medical Center for Cancer and Cardiovascular Diseases, The Center Institute Hospital of Japanese Foundation for Cancer Research, Tokushukai Hospitals and Tokyo Metropolitan Geriatric Hospital. The case group was composed of individuals registered as having T2D, while the control group was composed of those registered as not having T2D but with diseases other than T2D or being disease free.

For the GWAS, we included 7,360 Japanese individuals (4,449 T2D cases and 2,911 controls), for which we eliminated closely related subjects based on identity-by-descent (IBD), 180 individuals (21 T2D cases, 159 controls) without clinical risk factors (age, gender, Body-Mass Index; BMI) and two outliers by principal-component analysis (one T2D case, one control) (Figure S2) [37]. The subjects were genotyped using Illumina Human610-Quad and Illimina HumanHap550v3 BeadChips. The most popular and significant three clinical risk factors for which we could obtain information (age, gender and BMI) were used.

We excluded all SNPs with a genotype call rate <0.99, a Hardy-Weinberg equilibrium 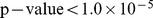
or a MAF <0.05. In total, 429,627 SNPs passed these stringent quality control criteria. To create a Manhattan plot of p-values from the GWAS, we used the Haploview 4.2 (http://www.broadinstitute.org/haploview). A QQ plot of p-values from the GWAS was created using the statistical software R [38].

### Cohort data for further validation

A population-based prospective study of cardiovascular disease and its risk factors has been underway since 1961 in the town of Hisayama, a suburb of the Fukuoka metropolitan area on the island of Kyushu, Japan. From national census and nutrition survey data, the age, occupational distributions, and nutritional intake of the population were almost identical to those of Japanese as a whole [39]. A validation cohort survey was conducted in the same town and in a similar fashion in 2002. The study design of the survey has been reported previously [40] [41]. Of the 3,896 residents aged 40–79 years, 3,000 consented to participate in a comprehensive assessment. Of them, 178 participants were not administered the oral glucose tolerance test: 100 subjects refused the test, 46 had already taken breakfast and the remaining 32 were receiving insulin therapy for diabetes. Consequently, 2,822 subjects completed the oral glucose tolerance test. A further 706 of the 2,822 subjects were excluded: 485 subjects were diagnosed with diabetes, 165 subjects had never participated in the comprehensive assessment for diabetes, 32 subjects refused gene tests and 24 subjects were not obtained genotype data. The remaining 2,116 subjects were determined to constitute the validation cohort.

### Algorithms for risk factor detection

Top-ranked SNPs were detected using three algorithms, the Cochran-Armitage test for trend, asymptotic Bayes factor (ABF) [26] and sure independence screening (SIS) [27], and with an additive association model for training set. We describe the details below.

The phenotype 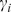 of subject 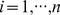 was set as the dependent variables (case = 1, control = 0) and the genotype 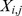 of each SNP 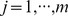 for a subject *i* as the independent variables with an additive association model (homozygous AA = 0, heterozygous AB = 1, homozygous BB = 2). Let 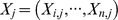 be all genotypes for a SNP *j*.

### Sure Independence Screening (SIS)

For each a set of genotypes 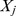 for a SNP *j*, we calculated the marginal utility
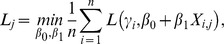
where 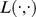 is a generic loss function for a logistic regression method defined by 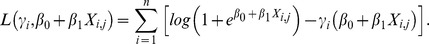



All SNPs were ranked according to the marginal utilities. The SNPs with the smaller 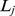 were indicated as more significant ones.

### Asymptotic Bayes Factor (ABF)

For each a set of genotypes 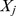 for a SNP *j*, the asymptotic Bayes factor (ABF) was calculated based on the output from a logistic regression method:
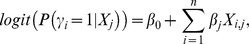
where 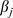 is the regression coefficient for the *j^th^* genetic variable. Let 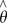 and 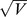 represent the maximum likelihood estimate (MLE) and standard error from the logistic regression method for a SNP *j*. The MLE 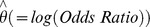 has then the normal distribution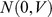 asymptotically when the sample size *n* is sufficiently large. Combining this likelihood with a normal prior 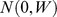 on the 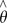, the asymptotic Bayes factor was calculated as below:
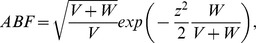
where 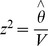 is the Wald statistic and *W* was set into 0.21^2^ corresponding to a 95% belief that the odds ratio is less than 1.5. This calculation was conducted using R code (http://faculty.washington.edu/jonno/BFDP.R). The SNPs with the smaller ABF were considered more significant.

We detected top-ranked SNPs using three algorithms as described above. In addition, we also considered cases with and without the r-square between SNPs. In the case of considering the r-square, we excluded any SNPs with r-square >0.8 from the predictors. The information of all r-squares between SNPs was obtained from the HapMap Japanese JPT population [42].

### Regression methods for risk model construction

We applied the ridge regression, the elastic net and the lasso method known as penalized regression methods. Let 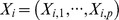 be the values of pre-selected top-ranked *p* SNPs for a subject *i* and let 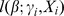 be the logistic log-likelihood:
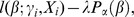
Where 
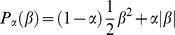
 [43] and 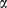 was set to 1 (the lasso), 0 (the ridge regression) and 0.1 to 0.9 at 0.1 intervals (the elastic net) and optimal penalty parameter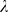 are selected using 10-fold cross-validation. Many coefficients 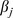 of the lasso and the elastic net methods are then set to 0. All penalty regression methods in this study were conducted using the *glmnet* package in the statistical software R [38].

### Evaluation of risk prediction models

All data were strictly separated into the training set and test set. For the training set, the top-ranked *p* SNPs were detected and selected stepwise using three algorithms and the r-square between SNPs information. Using a combination of *p* genetic risk factors (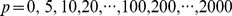) and clinical risk factors, risk prediction models were constructed based on three penalized regression methods. The selection of the risk prediction models was conducted based on 10-fold cross-validation against the training set; nine-tenths for top-ranked SNPs determination and model fitting and one-tenth for the validation. This process was repeated 10 times (10-fold cross validation). On the basis of the average AUC, we determined the optimal number of SNPs for model construction for each combination of algorithms and methods. Final models were constructed using the complete training set and the adjusted models were evaluated on the independent test set. The receiver operator characteristic (ROC) curves [44] on the test set and the area under the curve (AUC) were indicated as the discriminative accuracy of the risk prediction models.

### Detection of significant interaction factors

The significance of interaction factors was tested by comparing a logistic regression method including only the main effects to second method including the main effects as well as interaction factors that were composed of any combinations of genetic or clinical risk factors using a likelihood ratio test (i.e., deviation from a multiplicative model). The odds ratios (ORs), corresponding 95% confidence intervals (CIs) and p-values were calculated using statistical software R in order to determine the significance of the interaction factors [38]. We included interactions with p-values <0.05. By comparing the risk prediction model with and without including the interactions, we evaluated the significance of the interactions.

## Supporting Information

Figure S1 **A quantile-quantile (QQ) plot for association results for training set.**
(TIF)

Figure S2 **Relatedness among Japanese, Han Chinese, European and African individuals.** The two-dimensional plots with the first and the second components showed that 45 East Asian (HapMap populations of Japanese in Tokyo: jpt), 45 Han Chinese in Beijing: chb), 90 African (HapMap population of Yoruba in Ibadan, Nigeria: yri), 90 European (HapMap population of Utah, USA residents with ancestry from northern and western Europe: ceu) populations. Two outliers (case 1, control 1) were excluded from 4,450 cases and 2,912 controls.
(PDF)

Table S1 **Top-ranked 10 SNPs defined in ABF.**
(DOCX)

Table S2 **The top AUCs observed in elastic net method and the number of SNPs used in risk prediction model construction.**
(DOCX)
