# Supplementary figures and images for: The Construction of Risk Prediction Models Using GWAS Data and Its Application to a Type 2 Diabetes Prospective Cohort
Source: PLoS One. 2014 Mar 20;9(3):e92549. doi: 10.1371/journal.pone.0092549 (PMC3961382; doi:10.1371/journal.pone.0092549)

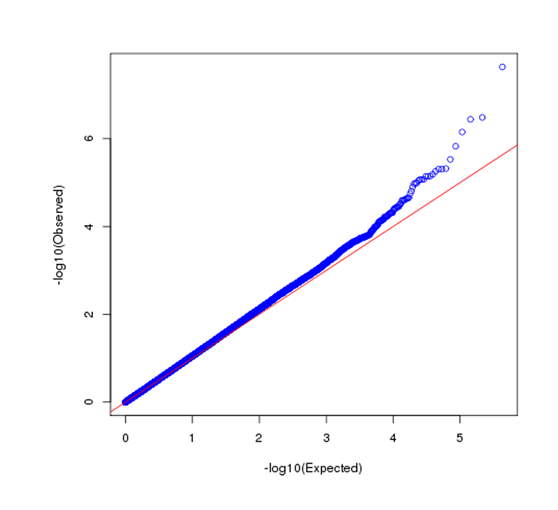

Supplement: Figure S1 — A quantile-quantile (QQ) plot for association results for training set. (TIF) [file pone.0092549.s001.tif]

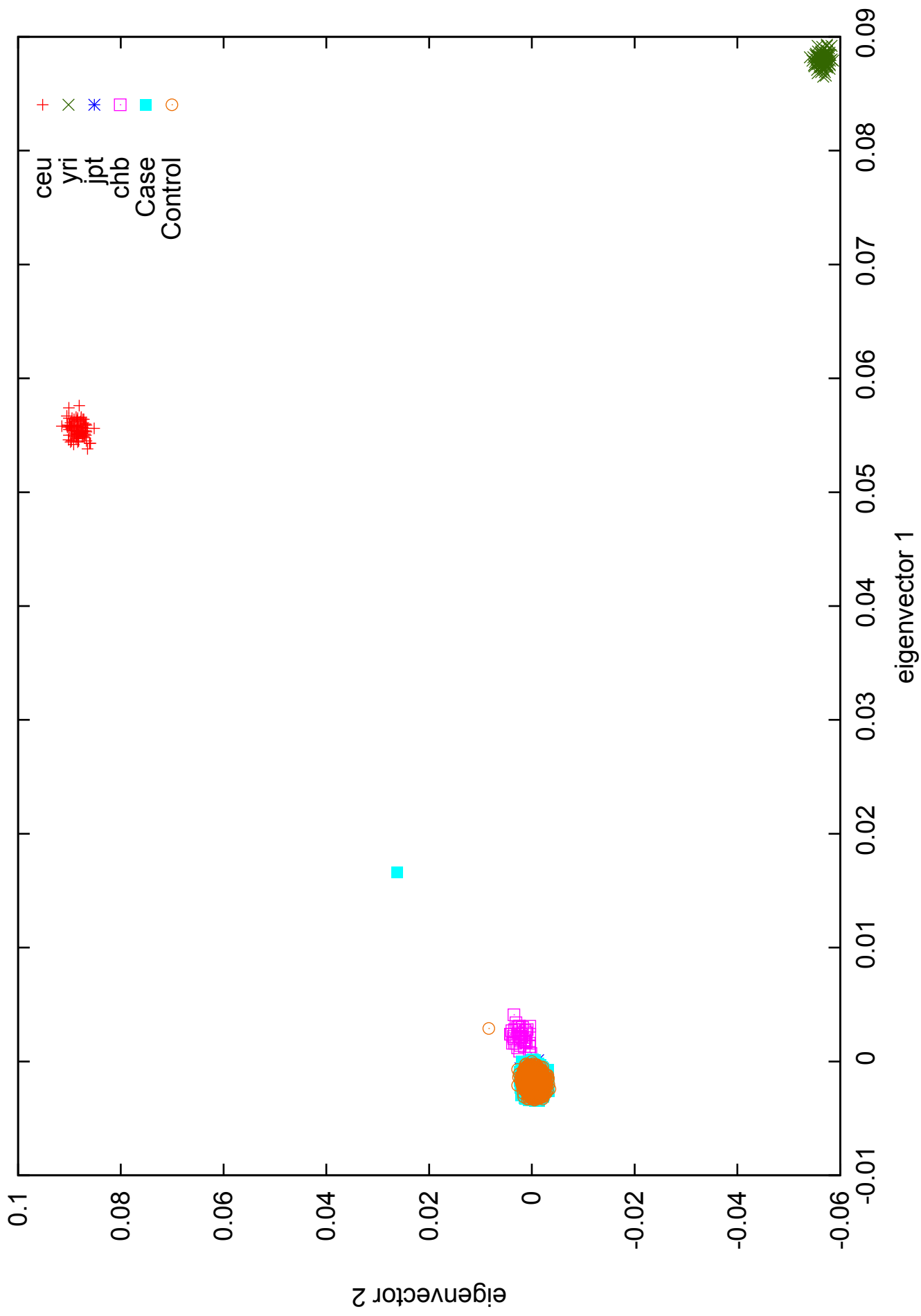

Supplement: Figure S2 — Relatedness among Japanese, Han Chinese, European and African individuals. The two-dimensional plots with the first and the second components showed that 45 East Asian (HapMap populations of Japanese in Tokyo: jpt), 45 Han Chinese in Beijing: chb), 90 African (HapMap population of Yoruba in Ibadan, Nigeria: yri), 90 European (HapMap population of Utah, USA residents with ancestry from northern and western Europe: ceu) populations. Two outliers (case 1, control 1) were excluded from 4,450 cases and 2,912 controls. (PDF) [file pone.0092549.s002.pdf]
