# Supplementary material for: The Construction of Risk Prediction Models Using GWAS Data and Its Application to a Type 2 Diabetes Prospective Cohort
Source: PLoS One. 2014 Mar 20;9(3):e92549. doi: 10.1371/journal.pone.0092549 (PMC3961382; doi:10.1371/journal.pone.0092549)
Supplement: Table S1 — Top-ranked 10 SNPs defined in ABF. (DOCX) [file pone.0092549.s003.docx]

**Table S1. Top-ranked 10 SNPs defined in ABF.**

A SNP rs12922855 (rank 3) was not used in our prediction model.

| Rank | rs # | MAF  (case) | MAF  (control) | Trend p-value | Chr. | Gene |
| --- | --- | --- | --- | --- | --- | --- |
| 1 | rs2237892 | 0.3415 | 0.3888 | $2.31\times10^{-8}$ | 11 | KCNQ1 |
| 2 | rs11865230 | 0.0226 | 0.0105 | $3.28\times10^{-7}$ | 16 | A2BP1 |
| 3 | rs12922855 | 0.0224 | 0.0105 | $3.63\times10^{-7}$ | 16 | A2BP1 |
| 4 | rs163171 | 0.4002 | 0.4437 | $7.06\times10^{-7}$ | 11 | KCNQ1 |
| 5 | rs11514706 | 0.4586 | 0.4988 | $1.49\times10^{-6}$ | 7 | DGKB |
| 6 | rs7901695 | 0.0571 | 0.0391 | $2.95\times10^{-6}$ | 10 | TCF7L2 |
| 7 | rs2328531 | 0.3540 | 0.3934 | $4.79\times10^{-6}$ | 6 | CDKAL1 |
| 8 | rs1436953 | 0.3769 | 0.4164 | $4.94\times10^{-6}$ | 15 | C2CD4A/B |
| 9 | rs2206734 | 0.4495 | 0.4091 | $5.54\times10^{-6}$ | 6 | CDKAL1 |
| 10 | rs4813894 | 0.4549 | 0.4946 | $7.16\times10^{-6}$ | 20 | PAK7 |
