# Supplementary material for: The Construction of Risk Prediction Models Using GWAS Data and Its Application to a Type 2 Diabetes Prospective Cohort
Source: PLoS One. 2014 Mar 20;9(3):e92549. doi: 10.1371/journal.pone.0092549 (PMC3961382; doi:10.1371/journal.pone.0092549)
Supplement: Table S2 — The top AUCs observed in elastic net method and the number of SNPs used in risk prediction model construction. (DOCX) [file pone.0092549.s004.docx]

**Table S2. The top AUCs observed in elastic net method and the number of SNPs used in risk prediction model construction.**

| algorithm | alpha used for elastic net | #SNPs used | AUC (combined)  95%CIs | AUC (combined) with r-square  95%CIs |
| --- | --- | --- | --- | --- |
| GWAS | 0.1 | 5 | 0.8025 (0.7689-0.8362) | 0.8025 (0.7689-0.8362) |
|  | 0.2 | 5 | 0.8026 (0.7690-0.8362) | 0.8026 (0.7690-0.8362) |
|  | 0.3 | 5 | 0.8025 (0.7689-0.8361) | 0.8025 (0.7689-0.8361) |
|  | 0.4 | 5 | 0.8026 (0.7690-0.8362) | 0.8026 (0.7690-0.8362) |
|  | 0.6 | 5 | 0.8026 (0.7690-0.8362) | 0.8026 (0.7690-0.8362) |
|  | 0.7 | 5 | 0.8026 (0.7691-0.8362) | 0.8026 (0.7691-0.8362) |
|  | 0.8 | 5 | 0.8027 (0.7691-0.8363) | 0.8027 (0.7691-0.8363) |
|  | 0.9 | 5 | 0.8027 (0.7691-0.8363) | 0.8027 (0.7691-0.8363) |
| SIS | 0.1 | 5 | 0.7993 (0.7655-0.8331) | 0.7989 (0.7651-0.8328) |
|  | 0.2 | 5 | 0.7992 (0.7654-0.833) | 0.7994 (0.7656-0.8332) |
|  | 0.3 | 5 | 0.7993 (0.7655-0.8331) | 0.7993 (0.7655-0.8331) |
|  | 0.4 | 5 | 0.7993 (0.7655-0.8331) | 0.7993 (0.7655-0.8331) |
|  | 0.6 | 5 | 0.7994 (0.7656-0.8332) | 0.7994 (0.7656-0.8332) |
|  | 0.7 | 5 | 0.7995 (0.7657-0.8332) | 0.7995 (0.7657-0.8332) |
|  | 0.8 | 5 | 0.7995 (0.7657-0.8332) | 0.7995 (0.7657-0.8332) |
|  | 0.9 | 5 | 0.7995 (0.7657-0.8333) | 0.7995 (0.7657-0.8333) |
| ABF | 0.1 | 10 | 0.8053 (0.7718-0.8388) | 0.8055 (0.7721-0.8388) |
|  | 0.2 | 10 | 0.8053 (0.7718-0.8388) | 0.8055 (0.7721-0.8388) |
|  | 0.3 | 10 | 0.8053 (0.7719-0.8388) | 0.8055 (0.7721-0.8388) |
|  | 0.4 | 10 | 0.8054 (0.7719-0.8389) | 0.8055 (0.7722-0.8389) |
|  | 0.6 | 10 | 0.8054 (0.7719-0.8389) | 0.8056 (0.7723-0.8390) |
|  | 0.7 | 10 | 0.8054 (0.772-0.8389) | 0.8057 (0.7723-0.8390) |
|  | 0.8 | 10 | 0.8055 (0.7721-0.8389) | 0.8057 (0.7723-0.8390) |
|  | 0.9 | 10 | 0.8054 (0.772-0.8389) | 0.8057 (0.7724-0.8391) |
